# Recurrent Coronary Artery Fistulae and a Novel Transforming Growth Factor Beta-3 Mutation

**DOI:** 10.7759/cureus.17780

**Published:** 2021-09-06

**Authors:** Naser Abdelhadi, Mohamed Zghouzi, Yasar Sattar, Maan Jokhadar, M. Chadi Alraies

**Affiliations:** 1 Internal Medicine, Emory University School of Medicine, Atlanta, USA; 2 Internal Medicine, Detroit Medical Center, Detroit, USA; 3 Internal Medicine, Icahn School of Medicine at Mount Sinai, New York, USA; 4 Cardiology, Detroit Medical Center, Detroit, USA

**Keywords:** loeys-dietz syndrome, tgfb3, e244k, arterial aneurysms

## Abstract

Loeys-Dietz syndrome (LDS) is a rare connective tissue disease associated with mutations in transforming growth factor (TGF) signaling leading to an increased risk of arterial calcification, aneurysms, and/or dissections. We report a case in which genetics evaluation revealed a rare variant E244K in the TGFB3 gene. The variant leads to the substitution of glutamic acid for lysine, two amino acids with dissimilar properties. Analysis from evolutionary data shows the glutamic acid is maintained across species. The clinical significance of the E244K variant in association with LDS was never previously reported as pathologic. This case report aims to report that the significance of the E244K variant in the TGFB3 gene is found to be pathologic in our case. A search on the Genome Aggregation Database (gnomAD) did not reveal any previously identified individuals with this variant, despite being a well-covered region. ClinVar has a few entries for E244K, where most of them are listed as unknown significance. Bringing together the genotype evidence with our patient’s clinical picture, we consider the variant to be pathogenic for this family.

## Introduction

Loeys-Dietz syndrome (LDS) is a rare connective tissue disease described by Belgian physician Bart L. Loeys and American physician Harry S. Dietz in 2005. According to the National Institutes of Health/National Library of Medicine, LDS prevalence is unknown, and out of five types, type 1 and 2 being the most common forms [1,2]. LDS is associated with mutations transforming growth factor (TGF) signaling. These mutations predispose the affected individuals to cardiovascular abnormalities, including arterial calcification, aneurysms, and dissections [1]. Other non-cardiovascular manifestations of the disease are craniofacial and skeletal abnormalities [3]. We describe a case of recurrent coronary dissection in a patient with a new TGF mutation.

## Case presentation

**Figure 1:**
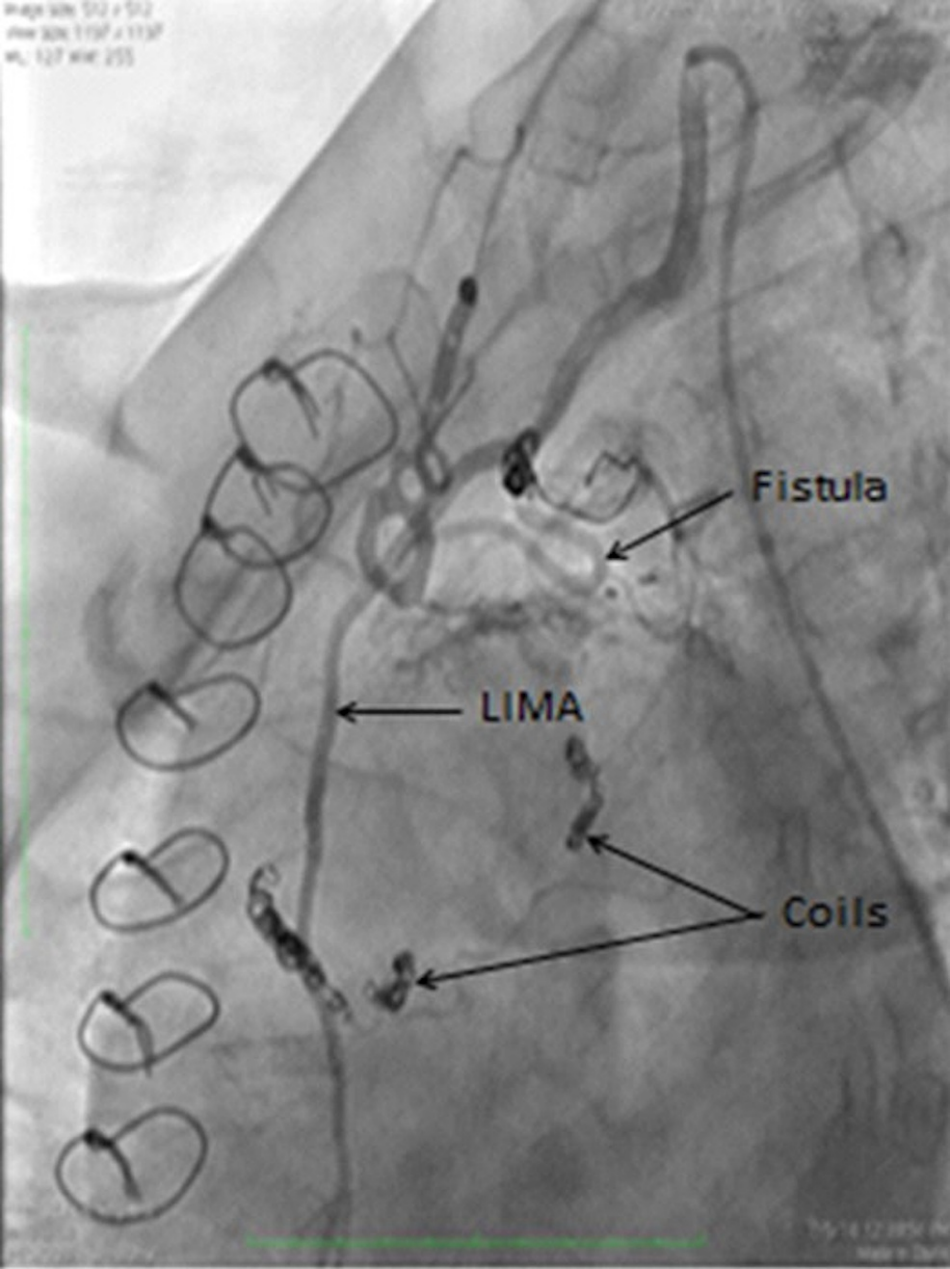
Shows a fistula between LIMA and pulmonary artery, which is strongly linked to the novel TGFB3 mutation detected in our case. This fistula was initially coiled and eventually surgically removed. LIMA, left internal mammary artery; TGFB3, transforming growth factor-beta 3.

**Figure 2:**
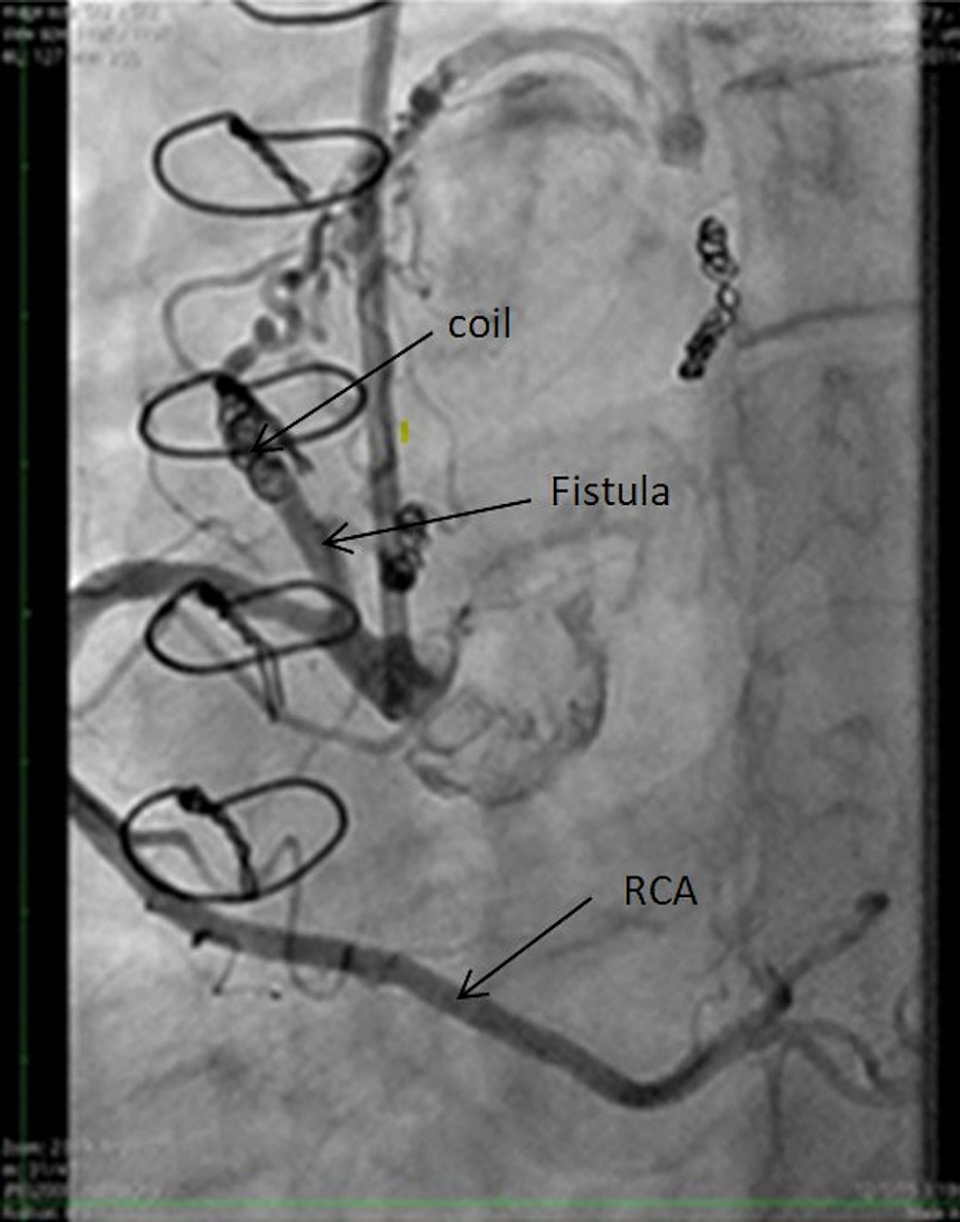
Shows another fistula between the RCA and the pulmonary artery, which is strongly linked to the novel TGFB3 mutation detected in our case. This fistula was initially coiled and eventually surgically removed. RCA, right coronary artery; TGFB3, transforming growth factor-beta 3.

A 61-year-old woman with a past medical history of heart failure and hypertension presented 10 years ago with a heart murmur and was found to have an atrial septal defect and also found to have several coronary artery fistulae on cardiac catheterization. These included fistulous connections between the left internal mammary artery (LIMA) to pulmonary artery (PA) (Figure 1) and between the right coronary artery (RCA) and PA (Figure 2). She subsequently underwent coiling of the fistulae between the LIMA to PA (Figure 1), RCA to PA (Figure 2), and left anterior descending to PA. She had recurrent symptoms within a few months, and fistulae recurrence was seen on cardiac catheterization. She then underwent surgical fistula ligation of RCA to PA (Figure 2), LIMA to PA (Figure 1), and right ventricle to PA. The PA was covered with a bovine pericardial patch to prevent a recurrence. In addition to coronary fistulae, our patient also had spontaneous right vertebral dissection that was managed conservatively. Additional observations included tortuous carotid arteries, persistent joint laxity, and atrophic scars. Her mother had an aortic aneurysm in her 50s without further details, and her family history was unremarkable for features of connective tissue disease. Genetics evaluation and testing revealed a novel E244K variant in the TGFB3 gene. Other potential causes of coronary artery fistula were ruled out. Therefore, the only likely cause of fistula in our case was the mutation in the TGFB3, which is strongly linked to LDS.

## Discussion

Our patient with recurrent coronary artery fistulae was found to have a heterozygous mutation in the gene that encodes TGFB3, a member of the TGF-beta superfamily of growth factor proteins that are involved in cellular proliferation and differentiation in many cell types [4,5]. We detected a novel heterozygous mutation in TGFB3, c.730G&gt>A, predicting the missense substitution p.Glu300Lys located on the long arm of chromosome 14 at position 24.3 [4]. Heterozygous pathogenic variants in TGFB3 are associated with LDS-5, characterized by features that overlap clinically with LDS-5 and Marfan syndromes, including growth abnormalities, cleft palate, pectus excavatum, joint hyperextensibility, and arachnodactyly [6]. Individuals with LDS-5 also have significant cardiac involvement, including thoracic/abdominal aortic aneurysm and dissection, and mitral valve disease [7].

The E244K variant is novel with previously unreported significance. The Genome Aggregation Database (gnomAD) search did not reveal any previously identified individuals with this variant. ClinVar has a few entries for E244K, where most of them are listed as unknown significance. The variant leads to the substitution of glutamic acid for lysine, two amino acids with different properties. Analysis from evolutionary data shows that the glutamic acid is maintained across species. This variant leads to a variety of clinical findings consistent with LDS. Bringing together the genotype evidence with our patient’s clinical picture, we consider the variant pathogenic.

## Conclusions

In the literature, there is a wide clinical variability associated with TGFB3 mutation. Our case highlights the presence of a rare E244K variant in the TGFB2 gene that is likely to be pathologic. To our knowledge, E244K was never previously reported.
